# Prognostic value of pretreatment systemic inflammatory markers in patients with locally advanced rectal cancer following neoadjuvant chemoradiotherapy

**DOI:** 10.1038/s41598-020-64684-z

**Published:** 2020-05-15

**Authors:** Yiyi Zhang, Xing Liu, Meifang Xu, Kui Chen, Shoufeng Li, Guoxian Guan

**Affiliations:** 1 Department of Colorectal Surgery, The First Affiliated Hospital of Fujian Medical University, Fuzhou, China; 2 Department of Colorectal Surgery, Fujian Medical University Union Hospital, Fuzhou, China; 3 Department of Pathology, Fujian Medical University Union Hospital, Fuzhou, China; 4 Department of General Surgery, The First Hospital of Fuzhou City Affiliated Fujian Medical University, Fuzhou, China

**Keywords:** Outcomes research, Surgical oncology

## Abstract

The aim of this study was to explore the most powerful systemic inflammation marker of survival in locally advanced rectal cancer (LARC) patients and construct prognostic  nomograms. A total of 472 LARC patients undergoing neoadjuvant chemoradiotherapy (NCRT) and radical surgery from 2011 to 2015 were included. The optimal cutoff points for the systemic immune-inflammation index (SII); and neutrophil-to-lymphocyte (NLR), platelet-to-lymphocyte (PLR), and monocyte-to-lymphocyte (MLR) ratios were calculated and determined by using the X-tile program. The cut-off values were 797.6. 2.3, 169.5, and 0.4, respectively. Cox regression analysis demonstrated that higher pathological TNM stage, the AJCC tumor regression grade, and the NLR level were significantly associated with increased overall survival and disease-free survival. High NLR level (≥ 2.3) was associated with higher pre-NCRT CA19–9 levels, lower hemoglobin, larger tumor size, and more lymph nodes retrieved (p = 0.012, p = 0.024, and p < 0.001; p < 0.001, respectively). High NRL scores were associated with poorer 5-year disease-free survival and overall survival (p < 0.001, and p < 0.001, respectively). Predictive nomograms and time-independent receiver operating characteristic (ROC) curve that included the NLR score group were superior to those without NLR scores. Higher NLR scores (≥2 0.3) were associated with poorer DFS and OS in LARC patients. In addition, NLR was identified as the most effective marker for systemic inflammation, and the prognostic value was further confirmed by time-dependent ROC analysis. More intense adjuvant treatment could be considered for higher NLR score patients with LARC following NCRT.

## Introduction

The standard of care for locally advanced rectal cancer (LARC) is neoadjuvant chemoradiotherapy (NCRT) followed by total mesorectal excision (TME). This strategy offers a higher probability of tumor downsizing and downstaging, increased tumor resectability, and better local tumor control^1–3^. However, patients show a wide variation in responses to NCRT and thus, different oncological outcomes. Currently, it remains difficult to accurately predict treatment outcomes for LARC patients after NCRT. The identification of reliable biomarkers for the oncologic outcomes is important to assist in risk-adapted treatment strategies and subsequent surveillance.

The systematic inflammatory response is involved in the development, progression, treatment response, and prognosis of many cancers, including prostate, breast, and colorectal cancers (CRC)^4–6^. Accumulating evidence has demonstrated an association of systematic inflammation and resistance to radiotherapy and chemotherapy in CRC^7–9^. The systematic inflammatory response can be reflected by hematological parameters, including the systemic immune-inflammation index (SII), the neutrophil-to-lymphocyte ratio (NLR), the platelet-to-lymphocyte ratio (PLR), and the monocyte-to-lymphocyte ratio (MLR). Several studies have revealed that the hematological inflammatory markers could be predictive markers for oncological outcomes and chemoradiotherapeutic responses in rectal cancer patients^4,10,11^. However, the use of combined markers of systematic inflammation in LARC patients after NCRT has not yet been fully investigated. Additionally, reports on the most effective marker for systemic inflammation in LARC patients after NCRT have been inconsistent.

To address the gap in the literature, the present study aimed to explore the most powerful systemic inflammation markers for survival outcomes in LARC patients and construct prognostic predictive nomograms.

## Patients and Method

### Patients

In this study, we retrospectively analyzed 472 LARC patients who underwent NCRT and radical resection between 2011 and 2015. The patient inclusion criteria and exclusion criteria were reported in our previous study^12,13^. Tumor staging was evaluated by digital rectal examination, colonoscopy, chest radiography or CT, abdominopelvic MRI, and transrectal ultrasound (ERUS). Preoperative radiation and concurrent chemotherapy were performed in accordance with our previous study. Surgery was performed 6–10 weeks after the end of radiation. Total mesorectal excision and high ligation of the inferior mesenteric artery were surgical techniques routinely performed at our institution. About one month after surgery, the patients received postoperative adjuvant chemotherapy for six months according to the NCCN guidelines^14^. The follow-up protocol was also conducted according to the NCCN guidelines^14^. Briefly, in the first three years, the patients were followed-up every three months, except for tumor recurrence examinations, then biannually for the next two years, and annually thereafter. The last follow-up cutoff date was December 31, 2018.

### Definitions

The pathological tumor regression grade (TRG)^15^ was used to evaluate the tumor response to NCRT. Pathologic complete response (pCR) was defined as no residual tumor cells in the resected specimen, including the primary site and lymph nodes. Venous blood samples were obtained within one week before NCRT. The systematic inflammatory markers were calculated using the following equations: SII = platelet count × (neutrophil count/lymphocyte count), NLR = neutrophil count/lymphocyte count, PLR = platelet count/lymphocyte count, and MLR = monocyte count/lymphocyte count.

### Statistical analysis

The Statistic Package for Social Science (SPSS, version 23.0) and the R software package version 3.5.1 were used to perform the statistical analyses. Chi-squared or Fisher’s exact test was used to assess the categorical variables. Continuous variables were assessed via the analysis of variance (ANOVA). The X-tile program (http://www.tissuearray.org/rimmlab/) was used to calculate and determine the best cutoff points for the SII, NLR, PLR, and MLR counts^16^. The Kaplan-Meier method and log-rank test were performed to evaluate the survival outcomes. The risk factors for overall survival (OS) and disease-free survival (DFS) were calculated by the Cox proportional hazards model. Based on the Cox regression model analysis, a nomogram was developed by using the R software. Time-dependent ROC curves were plotted to evaluate the performance of the nomogram. Statistical significance was defined as *P* < 0.05.

## Results

### Cutoff values for SII, NLR, PLR, and MLR

**Figure 1:**
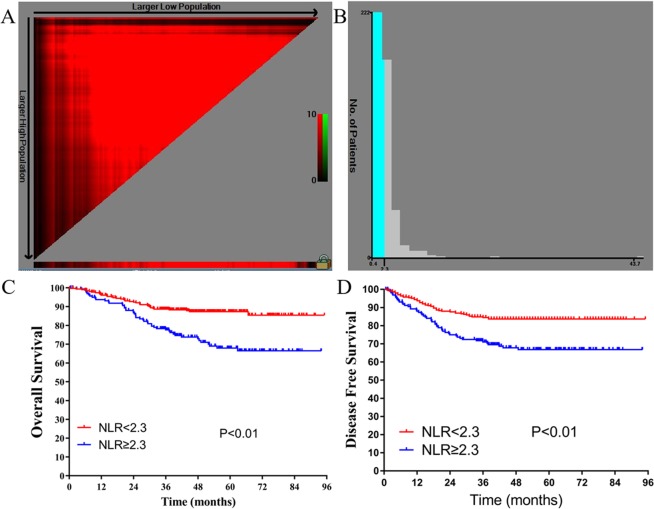
Cutoff points for NLR counts determined by the X-tile program. X-tile analysis divided the entire cohort into training sets (shown in the upper-left quartile of **A**) and matched validation sets (shown in the bottom X-axis of **A**) based on patient survival data. The black dot in the validation set represents the exact cutoff values for the NLR count. The entire cohort was divided into low (blue) and high (gray) NLR count groups based on the optimal cutoff point (2.3), as shown in a histogram of the entire cohort (**B**), a Kaplan-Meier curve of overall survival (**C**), and disease-free survival (**D**) for the optimal cutoff point of the NLR counts.

A total of 472 LARC patients (313 men and 159 women) were eligible for analysis in this study. The clinicopathological characteristics of the LARC patients are summarized in Supplementary Table 1. As seen in Fig. 1A,B, and Supplementary Figure 1, X-tile plots identified 797.6. 2.3, 169.5, and 0.4 as cutoff values for SII, NLR, PLR, and MLR, respectively. Based on the above cutoff points, we divided the entire cohort into low and high OS and DFS subgroups.

### Association of SII, NLR, PLR, and MLR with survival

**Figure 2:**
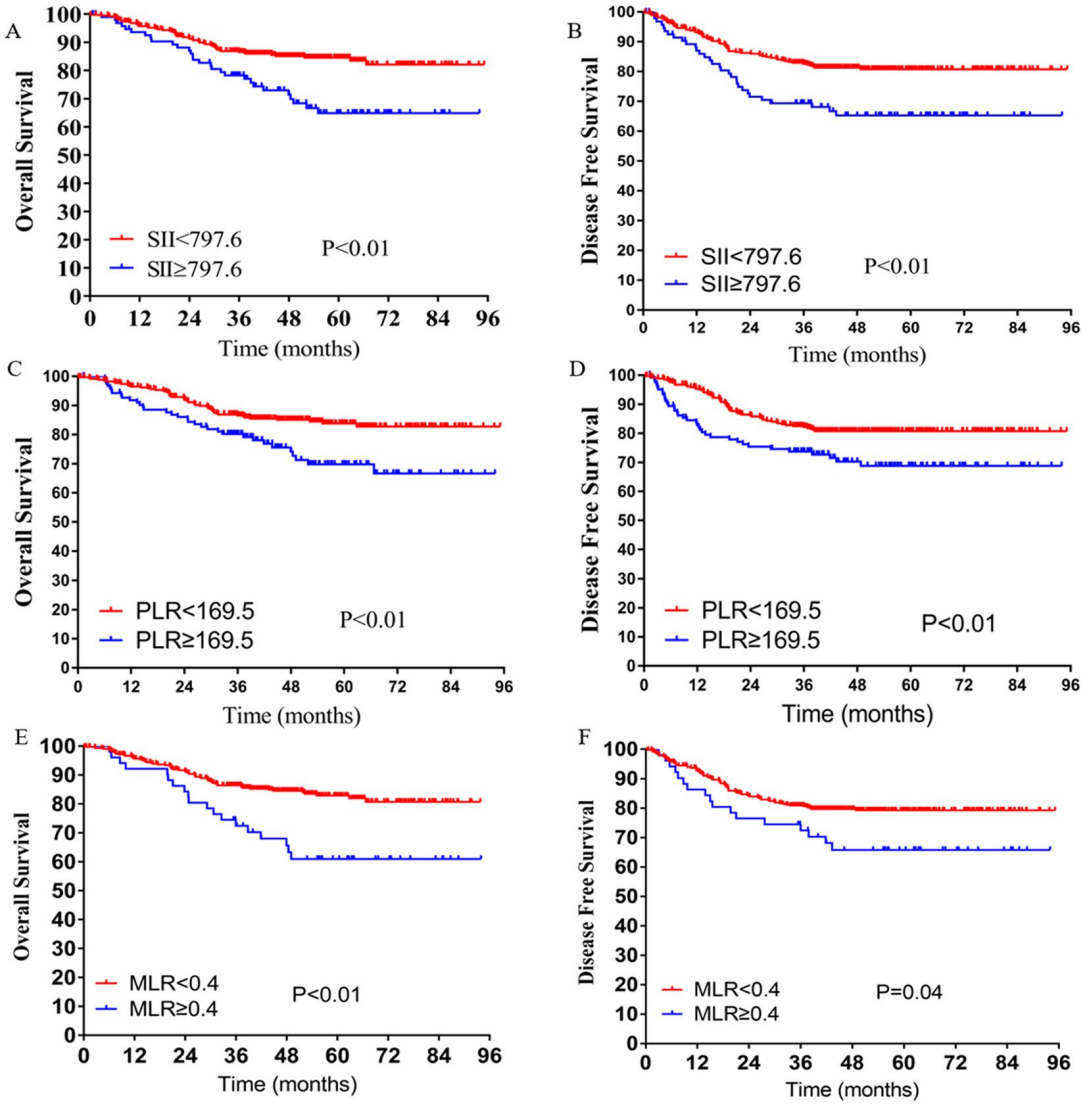
Kaplan-Meier analysis of the SII, PLR, and MLR counts. The overall survival (**A**) and disease-free survival (**B**) for the optimal cutoff point of the SII counts. The overall survival (**C)** and disease-free survival (**D**) for the optimal cutoff point of the PLR counts. The overall survival (**E**) and disease-free survival (**F**) for the optimal cutoff point of the MLR counts.

Higher SII, NLR, PLR, and MLR scores were correlated with worse prognosis in LARC patients following NCRT. The OS rates at three years for the low SII, NLR, PLR, and MLR groups were 86.5%, 88.7%, 86.5%, and 86.4%, respectively, significantly higher than 78.3%, 77.6%, 80.2%, and 72.4% in the high SII, NLR, PLR, and MLR groups, respectively (all *P* < 0.01, Figs. 1C and 2A,C, E). Notably, lower SII, NLR, PLR, and MLR scores were associated with better DFS, and the DFS rates at three years for the low SII, NLR, PLR, and MLR groups were 82.6%, 84.5%, 82.1%, and 80.9%, significantly higher than 69.3%, 71.0%, 73.7%, and 72.4% in the high SII, NLR, PLR, and MLR groups (*P* < 0.01, *P* < 0.01, *P* < 0.01, *P* = 0.04, respectively) (Figs. 1D and 2B,D, F).

### Prognostic value of SII, NLR, PLR and MLR

To explore the prognostic impact of SII, NLR, PLR, and MLR on OS and DFS in LARC patients, we performed a COX regression analysis. In the univariate analysis, tumor size (*P* < 0.001), pathological TNM stage (*P* < 0.001), AJCC grade (*P* < 0.001), pre-NCRT carcinoembryonic antigen (CEA) level (*P* = 0.025), pre-NCRT CA19–9 level (*P* < 0.001), anemia (*P* = 0.007), NLR level (*P* < 0.001), SII level (*P* = 0.001), MLR level (*P* = 0.001), PLR level (*P* = 0.004), and tumor differentiation (*P* < 0.001) were independently associated with OS in LARC patients following NCRT and TME (Table 1). Cox regression analysis demonstrated that the pathological TNM stage (HR = 1.777, 95%CI: 1.330–2.373, *P* < 0.001), AJCC grade (HR = 1.385, 95%CI: 1.013–1.894, *P* = 0.041), pre-NCRT CA19–9 level (HR = 1.731, 95%CI: 1.037–2.889, *P* = 0.036), and NLR level (HR = 1.797, 95%CI: 1.011–3.195, *P* = 0.046) were independent predictors of OS after NCRT, as shown in Table 1.

**Table 1:** Cox regression analysis of predictive factors for overall survival in patients with LARC following NCRT (n = 472).

Variables	Univariate analysis			Multivariate analysis		
	HR	95% CI	*P-value*	HR	95% CI	P- value
Sex, male/female	0.903	0.572–1.426	0.663			
Age	0.993	0.975–1.012	0.491			
ASA	1.110	0.715–1.722	0.642			
Distance from the anal verge	0.979	0.901–1.064	0.618			
Tumor size	1.447	1.275–1.642	<**0.001**	1.143	0.979–1.334	0.090
Pathological TNM stage	2.301	1.799–2.943	<**0.001**	1.777	1.330–2.373	<**0.001**
AJCC grade	2.190	1.691–2.835	<**0.001**	1.385	1.013–1.894	**0.041**
Interval time between NCRT and surgery	0.943	0.841–1.058	0.317			
Pre-NCRT cT stage	1.235	0.835–1.825	0.291			
Pre-NCRT cN stage	0.956	0.650–1.408	0.821			
Postoperative hospital stay	0.989	0.942–1.038	0.647			
Pre-NCRT CEA level	1.627	1.062–2.493	**0.025**	0.916	0.581–1.443	0.704
Pre-NCRT CA19–9 level	3.244	2.033–5.177	<**0.001**	1.731	1.037–2.889	**0.036**
Anemia	2.102	1.220–3.622	**0.007**	1.326	0.719–2.443	0.366
Hypoproteinemia	1.539	0.623–3.804	0.350			
NLR level	2.462	1.605–3.777	<**0.001**	1.797	1.011–3.195	**0.046**
SII level	2.122	1.355–3.323	**0.001**	0.895	0.472–1.696	0.733
MLR level	2.355	1.413–3.925	**0.001**	1.067	0.585–1.946	0.832
PLR level	1.897	1.226–2.935	**0.004**	1.169	0.672–2.035	0.581
Postoperative complications	1.151	0.625–2.120	0.651			
Neural invasion	1.911	0.833–4.383	0.126			
Vascular invasion	0.512	0.188–1.398	0.192			
Tumor differentiation	2.590	1.538–4.362	**<0.001**	1.180	0.675–2.063	0.560
Histopathology						
Expanding	Reference	Reference	0.645			
Infiltrating	0.638	0.157–2.594	0.530			
Ulcering	1.059	0.149–7.523	0.954			

LARC: locally advanced rectal cancer; NCRT, neoadjuvant chemoradiotherapy; HR, hazard ratio; CI, confidence interval; ASA: American Society of Anesthesiologists; AJCC: American Joint Committee on Cancer; CEA:carcinoembryonic Antigen; CA19–9: carbohydrate antigen 19–9; NLR: neutrophil-to-lymphocyte ratio; SII: systemic immune-inflammation index; MLR: monocyte-to-lymphocyte ratio; PLR: platelet-to-lymphocyte ratio.

On univariate analysis, tumor size (*P* < 0.001), pathological TNM stage (*P* < 0.001), AJCC grade (*P* < 0.001), pre-NCRT cT stage (*P* = 0.017), pre-NCRT CEA level (*P* = 0.011), pre-NCRT CA19–9 level (*P* < 0.001), anemia (*P* = 0.037), NLR level (*P* < 0.001), SII level (*P* = 0.001), PLR level (*P* = 0.004), neural invasion (*P* = 0.007), vascular invasion (*P* = 0.030), and tumor differentiation (*P* < 0.001) were independently associated with DFS in LARC patients following NCRT and TME (Table 2. Results from the Cox regression analysis demonstrated that the pathological TNM stage (HR = 1.573, 95%CI: 1.222–2.026, *P* < 0.001), AJCC grade (HR = 1.391, 95%CI: 1.038–1.864, *P* = 0.027), pre-NCRT cT stage (HR = 1.489, 95%CI: 1.018–2.179, *P* = 0.040), pre-NCRT CA19–9 level (HR = 1.707, 95%CI: 1.015–2.871, *P* = 0.047), and NLR level (HR = 1.707, 95%CI: 1.015–2.871, *P* = 0.044) were independent predictors of DFS after NCRT (Table 3.

**Table 2:** Operative and postoperative outcomes in patients with LARC following NCRT stratified by NLR.

Characteristics	NLR < 2.3 (n = 309)	NLR ≥ 2.3 (n = 163)	P-value
Operative time (min)	218.6 ± 53.2	228.0 ± 53.4	0.069
Estimated blood loss (ml)	91.9 ± 89.9	110.8 ± 136.6	0.072
Surgery approach (%)			0.890
Laparoscopic	219 (70.9)	114 (69.9)	
Open	90 (29.1)	49 (30.1)	
Pathological type (%)			0.214
Ulcering	296 (95.8)	161 (98.8)	
Expanding	7 (2.3)	1 (0.6)	
Infiltrating	6 (1.9)	1 (0.6)	
Histopathology (%)			0.134
Adenocarcinoma	285 (92.2)	143 (87.7)	
Mucinous or signet ring cell carcinoma	24 (7.8)	20 (12.3)	
Tumor differentiation (%)			0.157
Well-to-moderately differentiated	281 (90.9)	141 (86.5)	
Poorly differentiated and others	28 (9.1)	22 (13.5)	
Postoperative hospital stay (days)	8.75 ± 4.75	8.92 ± 4.58	0.709
Postoperative complications (%)	45 (14.6)	32 (19.6)	0.190
During CRT complications*(%)	132 (42.7)	62 (38.0)	0.376
Major	7 (2.3)	2 (1.2)	0.725
Organ preservation (%)	279 (90.3)	141 (86.5)	0.219
Lymph nodes retrieved	11.87 ± 5.84	14.27 ± 8.85	**<0.001**
Metastatic lymph nodes	0.718 ± 2.09	1.20 ± 3.55	0.063
CRM involvement (%)	1 (0.3)	1(0.6)	1.000
DRM involvement (%)	1 (0.3)	0	1.000
Tumor size (cm)	2.48 ± 1.22	3.15 ± 1.79	**<0.001**
Pathological TNM stage (%)			0.471
0	72 (23.3)	27 (16.6)	
I	74 (23.9)	42 (25.8)	
II	81 (26.2)	42 (25.8)	
III	79 (25.6)	50 (30.7)	
IV	3 (1.0)	2 (1.2)	
TRG (%)			0.188
0	72 (23.3)	27 (16.6)	
1	100 (32.4)	51 (31.3)	
2	110 (35.6)	72 (44.2)	
3	25 (8.1)	13 (8.0)	
pCR rates (%)	72 (23.3)	27 (16.6)	0.097
Neural invasion (%)	12 (3.9)	8 (4.9)	0.634
Vascular invasion (%)	12 (3.9)	2 (1.2)	0.153

LARC: locally advanced rectal cancer; NCRT, neoadjuvant chemoradiotherapy; NLR: neutrophil-to-lymphocyte ratio; CRM, circumferential resection margin; DRM, distal resection margin; TRG, tumor regression grade; pCR: pathologic complete response.

**Table 3:** Cox regression analysis of predictive factors for disease-free survival in patients with LARC following NCRT (n = 472).

Variables	Univariate analysis			Multivariate analysis		
	HR	95% CI	*P-value*	HR	95% CI	*P-value*
Sex, male/female	1.023	0.683–1.532	0.913			
Age	0.990	0.974–1.007	0.266			
ASA	1.054	0.703–1.582	0.798			
Distance from the anal verge	0.953	0.881–1.031	0.230			
Tumor size	1.347	1.196–1.517	**<0.001**	1.060	0.915–1.227	0.438
Pathological TNM stage	1.996	1.620–2.460	**<0.001**	1.573	1.222–2.026	**<0.001**
AJCC grade	2.042	1.619–2.575	**<0.001**	1.391	1.038–1.864	**0.027**
Interval time between NCRT and surgery	0.975	0.900–1.056	0.537			
Pre-NCRT cT stage	1.576	1.085–2.291	**0.017**	1.489	1.018–2.179	**0.040**
Pre-NCRT cN stage	1.331	0.618–2.867	0.465			
Postoperative hospital stay	0.990	0.948–1.034	0.649			
Pre-NCRT CEA level	1.651	1.123–2.427	**0.011**	1.040	0.686–1.577	0.852
Pre-NCRT CA19–9 level	2.687	1.722–4.191	**<0.001**	1.650	1.007–2.704	**0.047**
Anemia	1.762	1.034–3.002	**0.037**	1.230	0.689–2.195	0.484
Hypoproteinemia	1.240	0.505–3.044	0.639			
NLR level	2.075	1.412–3.047	**<0.001**	1.707	1.015–2.871	**0.044**
SII level	1.909	1.259–2.895	**0.002**	0.905	0.507–1.618	0.737
MLR level	1.623	0.965–2.729	0.068			
PLR level	1.622	1.083–2.430	**0.019**	1.175	0.708–1.950	0.532
Postoperative complications	1.158	0.670–2.000	0.600			
Neural invasion	2.546	1.285–5.045	**0.007**	1.047	0.473–2.315	0.911
Vascular invasion	0.401	0.176–0.914	**0.030**	0.605	0.234–1.568	0.301
Tumor differentiation	2.457	1.508–4.003	**<0.001**	1.264	0.744–2.148	0.286
Histopathology						
Expanding	Reference	Reference	0.217			
Infiltrating	0.502	0.159–1.583	0.240			
Expanding	1.093	0.220–5.415	0.914			

LARC: locally advanced rectal cancer; NCRT, neoadjuvant chemoradiotherapy; HR, hazard ratio; CI, confidence interval; ASA: American Society of Anesthesiologists; AJCC: American Joint Committee on Cancer; CEA:carcinoembryonic antigen; CA19–9: carbohydrate antigen 19–9; NLR: neutrophil-to-lymphocyte ratio; SII: systemic immune-inflammation index; MLR: monocyte-to-lymphocyte ratio; PLR: platelet-to-lymphocyte ratio

### Association of NLR with perioperative clinicopathological parameters

Among the patients included, 309 (65.5%) patients were categorized into the low-NLR group and 163 (34.5%) patients into the high-NLR group. Anemia and higher pre-NCRT CA19–9 levels were found in the high-NLR group (*P* < 0.05). No statistical differences were found between the two groups regarding gender, age, American Society of Anesthesiology (ASA) grade, the interval time between NCRT and surgery, the distance from the anal verge, clinical T stage, clinical N stage, hypoproteinemia, or pre-NCRT CEA level (Table 4).

**Table 4:** Baseline characteristics in patients with LARC following NCRT stratified by NLR.

Characteristics	NLR < 2.3 (n = 309)	NLR ≥ 2.3 (n = 163)	*P-value*
Sex (%)			0.124
Male	197 (64.6)	116 (66.4)	
Female	112 (35.4)	47 (33.6)	
Age (years)	56.29 ± 11.35	56.19 ± 11.69	0.925
ASA score (%)			0.718
1	237 (76.7)	123 (75.5)	
2	69 (22.3)	37 (22.7)	
3	3 (1.0)	3 (1.8)	
Distance from the anal verge (cm)	6.39 ± 2.43	6.73 ± 2.79	0.903
Interval time between NCRT and surgery (weeks)	9.00 ± 3.34	8.75 ± 3.31	0.777
Pre-NCRT cT stage (%)			0.214
T2	13 (4.2)	5 (1.2)	
T3	124 (40.1)	67 (41.1)	
T4	172 (55.7)	94 (57.7)	
Pre-NCRT cN stage (%)			0.863
N0	27 (8.7)	13 (8.0)	
N +	282 (91.3)	150 (92.0)	
Pre-NCRT CEA (%)			0.145
<5.0 ng/ml	181 (58.6)	84 (51.5)	
≥ 5.0 ng/ml	128 (41.4)	79 (48.5)	
Pre-NCRT CA19–9 (%)			**0.012**
<37.0 U/ml	275 (89.0)	131 (80.4)	
≥ 37.0 U/ml	34 (11.0)	32 (19.6)	
Anemia (%)	24 (7.8)	24 (14.7)	**0.024**
Hypoproteinemia (%)	10 (3.2)	11 (6.7)	0.100

NLR: neutrophil-to-lymphocyte ratio; LARC: locally advanced rectal cancer; NCRT: neoadjuvant chemoradiotherapy; ASA: American Society of Anesthesiologists; CEA:carcinoembryonic antigen; CA19–9: carbohydrate antigen 19–9

There were no significant differences between the groups regarding estimated blood loss, operation time, surgical approach, peri-NCRT complications, peri-NCRT major complications, and organ preservation procedures (Table 2). With regard to postoperative complications, no significant differences were seen between the two groups in terms of postoperative hospital stays and postoperative complications (*P* = 0.709, and *P* = 0.109, respectively).

Compared to the low-NLR group, the high-NLR group was associated with an increased number of lymph nodes retrieved (11.87 ± 5.84 vs 14.27 ± 8.85, *P* < 0.001). Moreover, the tumor size was larger in the high-NLR group (2.48 ± 1.22 vs 3.15 ± 1.79, *P* < 0.001). The high-NLR group tended to have lower pCR rates compared to the low-NLR group, but the difference was not significant. Pathological TNM stage, TRG, pathological type, histopathology, and tumor differentiation were similar in both groups (*P* = 0.471, *P* = 0.188, *P* = 0.214, *P* = 0.134, and *P* = 0.157, respectively). Similarly, neural invasion and vascular invasion did not differ between the two groups (*P* = 0.634, and *P* = 0.153, respectively).

### Predictive models for OS and DFS with/without NLR

**Figure 3:**
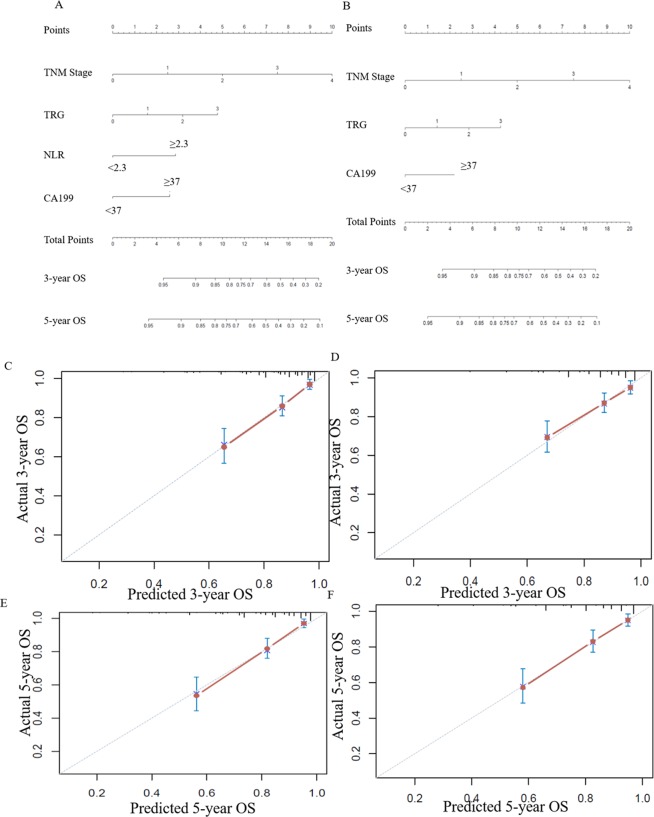
Construction of the factors for overall survival. (**A**) and (**B**) Nomograms developed for predicting overall survival, (**A**) the model with NLR counts, and (**B**) the model without NLR counts. (**C**) and (**E**) Calibration curves for 3- and 5-year OS for the model with NLR counts in LARC patients after NCRT with internal validation. (**D**) and (**F**) Calibration curves for 3- and 5-year OS for the model without NLR counts in LARC patients after NCRT with internal validation.

**Figure 4:**
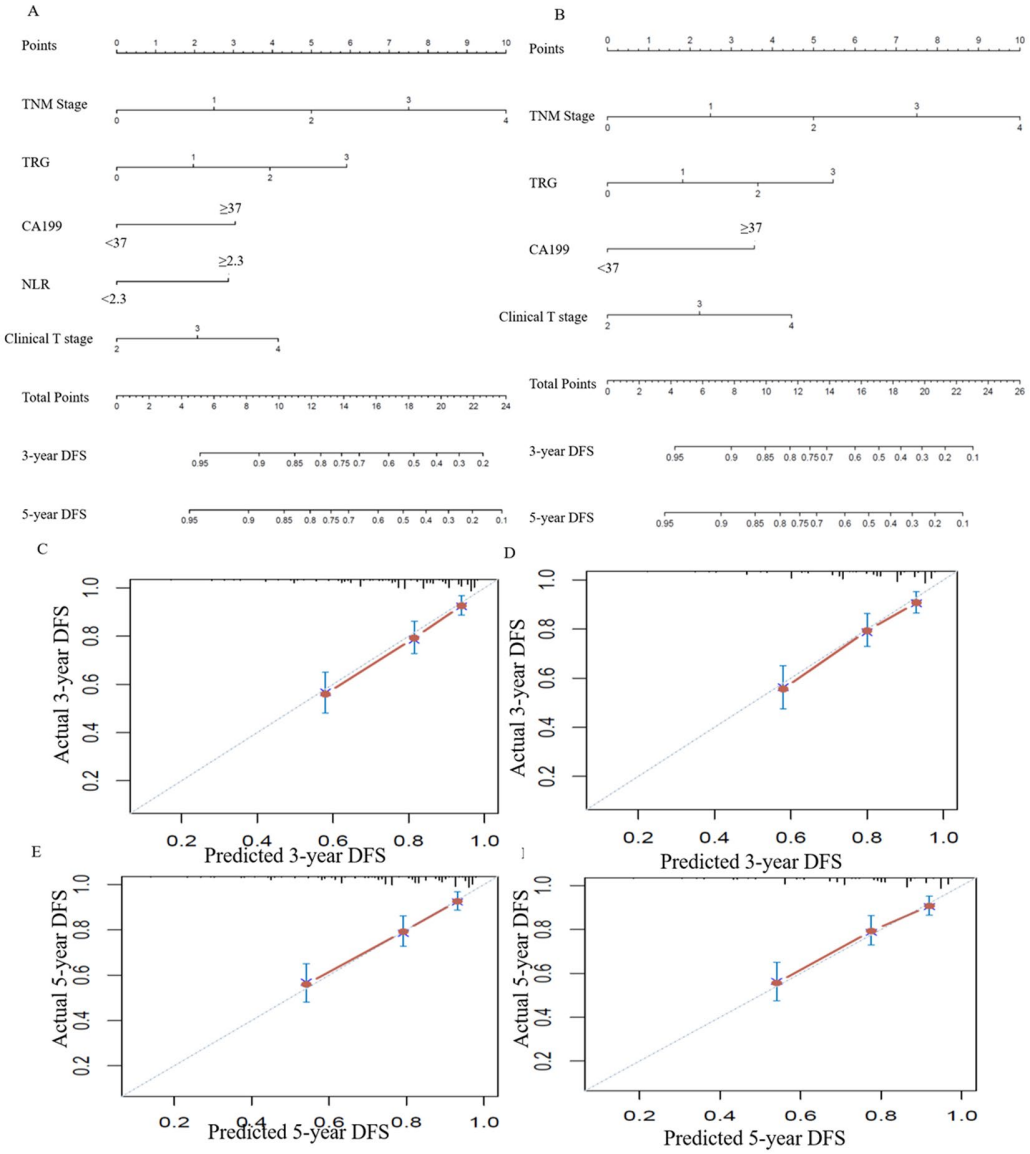
Construction of the factors for disease-free survival. (**A**) and (**B**) Nomograms developed for predicting disease-free survival, (**A**) the model with NLR counts, and (**B**) the model without NLR counts. (**C**) (**E**) Calibration curves for 3- and 5-year DFS for the model with NLR counts in LARC patients after NCRT with internal validation. (**D**) and (**F**) Calibration curves for 3- and 5-year DFS for the model without NLR counts in LARC patients after NCRT with internal validation.

Based on the above significant determinants, predictive nomograms for OS and DFS in LARC patients after NCRT were constructed (Figs. 3A and 4A). The 3-year OS and DFS predictive probabilities were obtained by drawing a straight line after summing up the score of each variable. Patients with a higher total score tended to have lower OS and DFS rates. The performance of the model was validated internally. The C-index of the nomogram including NLRs for predicting OS and DFS was 0.759 (95%CI: 0.707–0.816) and 0.737 (95%CI: 0.688–0.786), respectively. To further explore the role of the NLR in the predictive model, we constructed another model without NLRs (Figs. 3B and 4B). The C-index of the nomogram without NLRs for predicting OS and DFS was 0.741 (95%CI: 0.685–0.797) and 0.724 (95%CI: 0.719–0.729), respectively. The calibration curves showed good agreement between the predicted and actual probability of 3-, and 5-year OS (Fig. 3C,D, E, and F) and DFS (Fig. 4C,D, E, and F).

**Figure 5:**
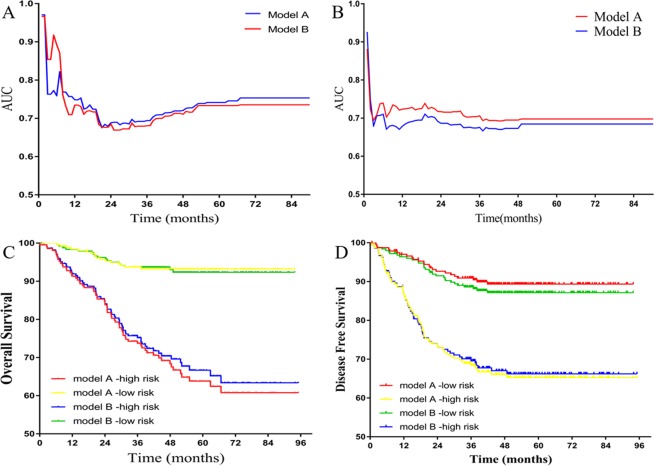
Comparing the effectiveness of the model with or without NLR counts. (**A**) Time-dependent AUC curves of the two models for predicting overall survival. (**B**) Time-dependent AUC curves of the two models for predicting disease-free survival. (**C**) Kaplan-Meier analysis of the two models for overall survival. (**D**) Kaplan-Meier analysis of the two models for disease-free survival. Model A was the model with NLR counts and Model B was the model without NLR counts. The risk score of each patient was calculated by the nomogram in each model and the patients were split evenly into the two groups.

The time-dependent ROC curves of the nomograms showed that all the areas under the curves (AUCs) were relatively stable after surgery during the observation period. However, the AUC of the model with NLRs tended to be higher than the model without NLRs at all times tested (Fig. 5A and B). To further evaluate whether the model with NLRs had a better predictive power for the prognosis of LARC patients, we calculated the prognosis of the two models using Kaplan-Meier survival analysis. The results showed that the model with NLRs had better discriminatory ability between the high and low-risk groups both in terms of OS and DFS.

## Discussion

Systematic inflammation is involved in the efficiency and toxicity of NCRT in rectal cancer patients. To our knowledge, few studies have evaluated the efficiency of using combined systematic inflammatory markers in LARC patients following NCRT. Herein, we showed that systematic inflammation evaluated by SII, NLR, MLR, and PLR, could act as an effective marker to predict the prognosis of LARC patients. Moreover, NLR was identified as the most effective marker for systemic inflammation, and the prognostic value was further confirmed by time-dependent ROC analysis. Finally, a nomogram was constructed to predict survival outcomes.

The association between inflammation and tumor biology was first reported by Virchow in 1863^17^. During the development of tumors, inflammation may promote cell mutagenesis, proliferation, and metastasis by generating high cytokine, reactive oxygen species (ROS), nitrogen, and tumor necrosis factor (TNF)-α, which are all involved in DNA damage. Additionally, pretreatment systemic inflammatory cellular activity may assist in the risk stratification for recurrence and survival in cancer patients^7,8^. The number of circulating lymphocytes, monocytes, neutrophils, and platelets are markers of immunologic response in CRC^18–22^. Systemic inflammatory indexes, such as LMR, NLR, SII, and PLR have been validated as indicators of ongoing systemic inflammation and worse outcomes in CRC patients^4,8,23,24^. Herein, we ascertained the prognostic implication of LMR, NLR, SII, and PLR, which was consistent with present findings.

The most effective marker for systemic inflammation in LARC patients after NCRT has not been conclusively demonstrated in previous studies. To explore the most effective marker for systemic inflammation, COX regression analysis was performed by using a combination of the above mentioned systemic inflammatory indexes. The Cox regression analysis identified NLR as the most effective marker representing systemic inflammation in LARC patients following NCRT. NLR is one of the most widely used biomarkers for systemic inflammation. The prognostic significance of NLR has been explored in a variety of cancers. NLR has been correlated with impaired oncological outcomes in patients with non-metastatic CRC^25,26^. In addition, NLR was reported to be an independent predictor of chemotherapeutic response in CRC patients^27,28^. However, the predictive role of NLR in LARC patients remains unclear. Jung et al^29^. reported that pre-CRT NLR was not able to distinguish recurrence-free patients with rectal cancer receiving NCRT. Herein, we demonstrated that NLR was the most effective marker for predicting OS and DFS in LARC patients. The discrepancy could be explained by the small sample size or racial differences. In addition, we revealed that higher NLRs correlated with increased numbers of lymph nodes retrieved and larger tumor size.

To further explore the prognostic significance of NLR in LARC patients, we developed two predictive models, constructed with and without NLR. The results demonstrated that the model containing NLRs was more powerful than the model without NLRs in predicting the DFS and OS of LARC patients. Our results further validated that NLRs have an important role in predicting DFS and OS in LARC patients following NCRT. In summary, in the present study, we successfully established a nomogram model to predict the outcomes of LACR patients and further confirmed that NLR played an indispensable role in the nomogram model.

Several limitations warrant discussion. First, the present study was subject to potential selection bias due to the retrospective design. Second, peripheral blood cell analysis results might be affected by factors such as blood circulation capacity, infection, and nutritional status. It is reasonable to have different blood cell analysis results and SII, NLR, MLR, and NLR results among different cohorts. Third, the impact of gene profiling and tumor microenvironment inflammation was not assessed owing to the lack of complete medical records. Despite these limitations, we believe that this study adds to the understanding of the impact of systemic inflammation on the oncological outcomes in patients with LARC following NCRT.

In conclusion, higher NLR scores (≥ 2.3) were associated with poorer DFS and OS in LARC patients. In addition, NLR was identified as the most effective marker for systemic inflammation, and the prognostic value was further confirmed by time-dependent ROC analysis. Finally, a nomogram was constructed to predict survival outcomes. More intense adjuvant treatment could be considered for higher NLR-score patients with LARC following NCRT. Larger-scale prospective clinical trials are warranted to confirm the above findings.

### Ethics approval and consent to participate

All subjects gave their informed consent for inclusion before they participated in the study. The study was conducted in accordance with the Declaration of Helsinki, and the protocol was approved by the Ethics Committee of Fujian Medical University Union Hospital.

## Supplementary information

Supplementary Information.

## Data Availability

The data generated or analyzed during this study are available from the corresponding author upon reasonable request.

## References

[1] Roh MS (2009). Preoperative multimodality therapy improves disease-free survival in patients with carcinoma of the rectum: NSABP R-03. J. Clin. Oncol..

[2] Sauer R (2012). Preoperative versus postoperative chemoradiotherapy for locally advanced rectal cancer: results of the German CAO/ARO/AIO-94 randomized phase III trial after a median follow-up of 11 years. J. Clin. Oncol..

[3] van Gijn W (2011). Preoperative radiotherapy combined with total mesorectal excision for resectable rectal cancer: 12-year follow-up of the multicentre, randomised controlled TME trial. Lancet Oncol..

[4] Jones HG (2018). Inflammatory cell ratios predict major septic complications following rectal cancer surgery. Int J Colorectal Dis.

[5] Karki R, Man SM, Kanneganti TD (2017). Inflammasomes and Cancer. Cancer Immunol Res.

[6] Sciarra A (2016). Prognostic value of inflammation in prostate cancer progression and response to therapeutic: a critical review. J Inflamm (Lond).

[7] Stotz M (2014). The preoperative lymphocyte to monocyte ratio predicts clinical outcome in patients with stage III colon cancer. Br. J. Cancer.

[8] Szkandera J (2014). The elevated preoperative platelet to lymphocyte ratio predicts decreased time to recurrence in colon cancer patients. Am. J. Surg..

[9] Wu Q, Hu T, Zheng E, Deng X, Wang Z (2017). Prognostic role of the lymphocyte-to-monocyte ratio in colorectal cancer: An up-to-date meta-analysis. Medicine (Baltimore).

[10] Shen J (2017). Prognostic Role of Neutrophil-to-Lymphocyte Ratio in Locally Advanced Rectal Cancer Treated with Neoadjuvant Chemoradiotherapy. Med. Sci. Monit..

[11] Zhang X (2019). Association of markers of systemic and local inflammation with prognosis of patients with rectal cancer who received neoadjuvant radiotherapy. Cancer Manag Res.

[12] Zhang Y, Sun Y, Xu Z, Chi P, Lu X (2017). Is neoadjuvant chemoradiotherapy always necessary for mid/high local advanced rectal cancer: A comparative analysis after propensity score matching. Eur J Surg Oncol.

[13] Sun Y (2018). Prognostic significance of neoadjuvant rectal score in locally advanced rectal cancer after neoadjuvant chemoradiotherapy and construction of a prediction model. J Surg Oncol.

[14] Benson AB (2018). Rectal Cancer, Version 2.2018, NCCN Clinical Practice Guidelines in Oncology. J Natl Compr Canc Netw.

[15] Ryan R (2005). Pathological response following long-course neoadjuvant chemoradiotherapy for locally advanced rectal cancer. Histopathology.

[16] Camp RL, Dolled-Filhart M, Rimm DL (2004). X-tile: a new bio-informatics tool for biomarker assessment and outcome-based cut-point optimization. Clin. Cancer Res..

[17] Balkwill F, Mantovani A (2001). Inflammation and cancer: back to Virchow. Lancet.

[18] Shibutani M (2017). The peripheral monocyte count is associated with the density of tumor-associated macrophages in the tumor microenvironment of colorectal cancer: a retrospective study. BMC Cancer.

[19] Chen JH (2017). Systemic immune-inflammation index for predicting prognosis of colorectal cancer. World J. Gastroenterol..

[20] Iseki Y (2017). The impact of the preoperative peripheral lymphocyte count and lymphocyte percentage in patients with colorectal cancer. Surg. Today.

[21] Rao XD (2018). Poor prognostic role of the pretreatment platelet counts in colorectal cancer: A meta-analysis. Medicine (Baltimore).

[22] Tanio A (2019). A prognostic index for colorectal cancer based on preoperative absolute lymphocyte, monocyte, and neutrophil counts. Surg. Today.

[23] Zhong JH, Huang DH, Chen ZY (2017). Prognostic role of systemic immune-inflammation index in solid tumors: a systematic review and meta-analysis. Oncotarget.

[24] Ward WH (2018). Predictive Value of Leukocyte- and Platelet-Derived Ratios in Rectal Adenocarcinoma. J. Surg. Res..

[25] Zhang J, Zhang HY, Li J, Shao XY, Zhang CX (2017). The elevated NLR, PLR and PLT may predict the prognosis of patients with colorectal cancer: a systematic review and meta-analysis. Oncotarget.

[26] EMC F (2017). Association of Systemic Inflammation and Sarcopenia With Survival in Nonmetastatic Colorectal Cancer: Results From the C SCANS Study. JAMA Oncol.

[27] Kim IH (2018). Clinical significance of changes in systemic inflammatory markers and carcinoembryonic antigen levels in predicting metastatic colorectal cancer prognosis and chemotherapy response. Asia Pac J Clin Oncol.

[28] Wu Y (2016). Neutrophil-to-lymphocyte and platelet-to-lymphocyte ratios predict chemotherapy outcomes and prognosis in patients with colorectal cancer and synchronous liver metastasis. World J Surg Oncol.

[29] Jung SW (2017). Association of immunologic markers from complete blood counts with the response to preoperative chemoradiotherapy and prognosis in locally advanced rectal cancer. Oncotarget.

