# Supplementary material for: Prognostic value of pretreatment systemic inflammatory markers in patients with locally advanced rectal cancer following neoadjuvant chemoradiotherapy
Source: Sci Rep. 2020 May 15;10:8017. doi: 10.1038/s41598-020-64684-z (PMC7228917; doi:10.1038/s41598-020-64684-z)
Supplement: Supplementary file 1 — Supplementary Information. [file 41598_2020_64684_MOESM1_ESM.pdf]

Prognostic value of pretreatment systemic inflammatory markers in patients with locally advanced rectal cancer following neoadjuvant chemoradiotherapy

Yiyi Zhang*1, Xing Liu*1, Meifang Xu*2, Kui Chen3, Shoufeng Li1, Guoxian Guan[#]1

*These authors contributed equally to this work

1 Department of Colorectal Surgery, The First Affiliated Hospital of Fujian Medical University, Fuzhou, China

2 Department of Pathology, Fujian Medical University Union Hospital, Fuzhou, China

3 Department of General Surgery, The First Hospital of Fuzhou City Affiliated Fujian Medical University， Fuzhou, China

Supplement Fig. 1 Cutoff points for PLR, MLR and SII counts determined by X-tile program.

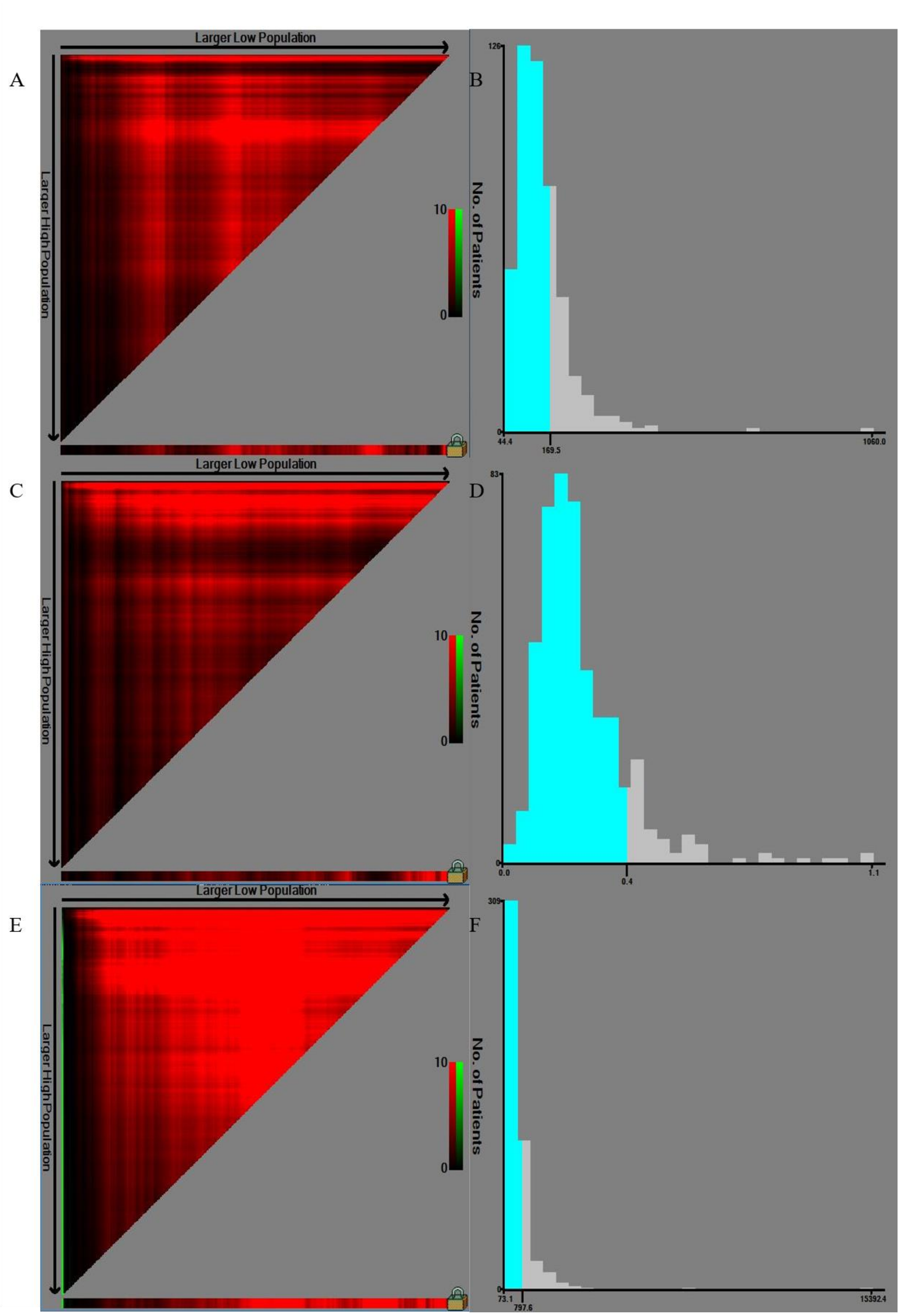

Supplemental Table 1 Baseline characteristic in patients with LARC following NCRT

**Characteristics**	**N (472)**	**%**
Sex (%)		
Male	313	66.3
Female	159	33.7
Age (years)	56.2 ± 11.4	
ASA score (%)		
1	360	76.3
2	106	22.5
3	6	1.3
Distance from the anal verge (cm)	6.5 ± 2.5	
Interval time between NCRT and surgery (weeks)	8.9±3.3	
Pre-NCRT cT stage (%)		
T2	15	3.2
T3	191	40.5
T4	266	56.4
Pre-NCRT cN stage (%)		
N0	40	8.5
N+	432	91.5
Pre-NCRT CEA (%)		
<5.0 ng/ml	265	56.1
≥5.0 ng/ml	207	43.9
Pre-NCRT CA19-9 (%)		
<37.0 ng/ml	406	86.0
≥37.0 ng/ml	66	14.0
Anemia (%)	48	10.2
Hypoproteinemia (%)	21	4.4

LARC: locally advanced rectal cancer; NCRT: neoadjuvant chemoradiotherapy; ASA: American Society of Anesthesiologists; CEA:Carcinoembryonic Antigen; CA19-9: Carbohydrate Antigen 19-9
